# The Matron's Department

**Published:** 1905-08-12

**Authors:** 


### HOSPITAL ADMINISTRATION.

### CONSTRUCTION AND ECONOMICS.

### THE MATRON'S DEPARTMENT.

#### II. WHAT MAY BE REQUIRED OF THE MATRON.

The word "matron" is as elastic as the word "nurse" and the one serves to cover as many degrees of work and status as the other. Even narrowing it in this connection to stand for "superintendent of nurses" the post of matron embraces so wide a diversity of duties, comparing one hospital with another, that there is hardly any branch of administrative work which the candidate may not find herself called upon to undertake. The nurse's career, as briefly sketched in a previous article, affords no formal training with regard to most of these duties, and it is therefore all the more necessary that those who look forward to matronships should seize every opportunity as it offers of getting the experience and skill they will one day need. Some of these duties may be enumerated but many defy analysis.

1. Superintendence of Nursing.

All the previous training and experience of the matron have been directed to this end. The years spent in good hospitals will have taught her how things ought to be done and should have made the work of keeping the nursing on a high level perfectly familiar to her. She will find it essential, however, to go on learning and to keep abreast of the times, unless she is prepared to find as years go on her supremacy questioned and her dicta politely doubted.

2. The Superintendence of Nurses.

Almost from the moment of entering the hospital the probationer has opportunities for learning to grapple with the great administrative problem "how to make other people work." Starting perhaps by teaching some raw beginner to fold a sheet she passes in her second year to definite duties towards the newcomers and so to the more responsible instructions of the staff nurse. At every stage, long before she is in a position of assured authority as ward sister, she has people to work under her, and more helpful still, she is working at the same time under others and can learn by personal observation the right and the wrong method of giving orders. There is no doubt that to get people to do their best is a gift of nature. But it is also an art which can be learned by any intelligent woman. There is no better place than a hospital in which to learn the art of government. Every hour gives opportunity for the self-restraint, patience and courage in dealing with subordinates, for loyalty and sincerity in obeying superiors, for reticence and the exercise of a judgment unbiassed by personal slights or favours. Of these materials are built the great foundation of justice. The probationer is no child at school. She is a woman in the prime of life preparing herself to have the issues of life or death entrusted to her. From the very first, right through her career she must keep in view the great principle of justice on which all sound government rests. Unless that is mastered, practically as well as theoretically, all the other attainments of the matron will be wasted; she can never be a success as the head of any institution. If justice be once grounded in the heart of the nurse, there will be no need for anxiety when she becomes the superintendent.

3. Teaching.

The duty of lecturing is a severe strain on many matrons admirably qualified in other respects for their work. What matron has not experienced that deadly sinking of the heart as the hour approaches when the long row of caps, aprons, and note-books is to be confronted? Sometimes this fear arises for want of the thorough knowledge of the subject which gives confidence to a teacher. But it is more often a diffidence about the power of expression, which results from want of practice in teaching.

Sound knowledge such as should inspire confidence alike in the teacher and the pupil is seldom to be gained merely by attendance at lectures during the probationer period. For one who is being prepared to lecture these opportunities of learning must be largely supplemented by additional instruction and by private study. When once the knowledge is gained there need be no difficulty in the way of any ward sister getting practice in teaching if she desires it. By coaching her own probationers, by repeating a lecture to some who have missed it, by stepping in when wanted to take the place of the usual teacher, she will find her powers of thinking and speaking clearly expand, and thus when placed in a position of authority she will be able to speak collectively to her nurses without causing discomfort to herself or others.

4. Superintendence of Servants.

The best training which a matron can have in the superintendence of the servant is what she gained as ward sister in the direction of her ward-maid. She will get in her own ward plenty of exercise in the art of making the best of indifferent material. Is she always complaining of the omissions, rudeness, and stupidity of the maid who falls to her share? Be sure that she has yet to learn the A B C of domestic management. On the other hand, the sister who has learned to explain clearly, to correct kindly and repeatedly, and to penetrate resistance by imperturbable good humour is on the road to become the mistress of a contented and efficient staff of servants.

5. Housekeeping.

It is unfortunately true that a nurse may remain many years in large hospitals without getting the slightest insight into the working of the household at large. There are some wide-minded matrons who afford their sisters in turn opportunities of learning housekeeping, but these are few in number. There is no such thing to be had as a practical course of institutional housekeeping. The best preparation available is to work as assistant matron, but then the number of assistant matronships is very small. It is an undoubted drawback to the smooth working of institutional households that new matrons are compelled, each in succession, to make their own experience out of their failures.

### 6. Accounts.

In most small hospitals the matron has to keep the books and is responsible for a large proportion of the accounts. If she has never had more to do with figures than the keeping of private accounts, this will probably prove a very irksome if not ill-discharged duty. If, however, a course of housekeeping is hard to obtain, the same cannot be said of book-keeping. No woman who aspires to the post of matron should neglect the precaution of taking a dozen lessons in this simple art. When it is properly understood there is a fascination even to those who are naturally unbusiness-like in well-regulated figures, but to have to find it all out for herself, perhaps after everyone else has gone to bed, and with weary expenditure of ink and sighs, is a process to daunt the clearest head.

### 7. Secretarial Work.

It is not usual for much clerical or secretarial work to be demanded of a matron. But it is possible she may have to take the minutes occasionally at meetings, and a knowledge of the procedure on such occasions will be of great service to her. The best possible way of learning the unwritten laws which govern committees is to follow attentively the course of proceedings at all formal meetings which may be attended. Any business friend could explain the procedure, and every fresh light thrown on the subject will add to its interest. It is to be regretted that women, as a rule, are completely unversed in the exact and ancient customs regulating meetings, since nearly all the public business of this country—from the House of Commons to the grammar school football club—is carried on through the medium of committees and under the protection of the same code of regulations.

### 8. Supervision of Private Nurses.

Finally, it is not unusual in provincial hospitals for the matron to undertake the duties of sending out and supervising the staff of private nurses attached to the institution. Personal knowledge of the pitfalls of private nursing is hardly to be expected of her, and in this branch of her duties the matron-elect must buy her own experience. With the watchword of *Justice* before her she need not fear to pay too high a price.

We have said that many duties will be required of the matron which defy analysis. For these there can be no formal preparation other than the preparation which every day of youth affords for the heavy responsibilities which await the woman in charge of a household, be she wife and mother or matron and superintendent of nurses.

